# The use of intercultural interpreter services at a pediatric emergency department in Switzerland

**DOI:** 10.1186/s12913-022-08771-z

**Published:** 2022-11-17

**Authors:** Sina Buser, Noemi Gessler, Myriam Gmuender, Ursula Feuz, Anne Jachmann, Jabeen Fayyaz, Kristina Keitel, Julia Brandenberger

**Affiliations:** 1 Division of Pediatric Emergency Medicine, Department of Paediatrics, Inselspital, Bern University Hospital, University of Bern, Bern, Switzerland; 2 Emergency Department, Inselspital, Bern University Hospital, University of Bern, Bern, Switzerland; 3 Division of Pediatric Emergency Medicine, Hospital for Sick Children, Toronto, Ontario Canada; 4 Edwin S.H. Leong Centre for Healthy Children, University of Toronto, Toronto, ON Canada

**Keywords:** Pediatric migrant health, Equity, Interpreter services, Communication in health care, Limited language proficiency, Pediatrics

## Abstract

The aim of our study was to analyze the use of interpreter services and improve communication during health encounters with families with limited language proficiency (LLP) at the pediatric emergency department (ED) of the University Hospital of Bern.

This study is a pre- and post-intervention study analyzing the use of interpreter services for LLP families. All families originating from a country with a native language other than German, English or French presenting to the ED were eligible to participate in the study. If they agreed to participate, the language proficiency of the caregiver present during the health encounter was systematically assessed during a phone interview within a few days after the consultation, using a standardized screening tool. If screened positive (relevant LLP), a second phone interview with an interpreter was conducted. Further variables were extracted including nationality, age, gender and date of visit using administrative health records. To increase the use of interpreter services, a package of interventions was implemented at the department during 3 months. It consisted of: i) in person and online transcultural teaching ii) awareness raising through the regular information channels and iii) the introduction of a pathway to systematically identify and manage LLP families.

The proportion of LLP families who received an interpreter was 11.0% (14/127) in the pre-intervention period compared to 14.8% (20/135) in the post-intervention period. The interpreter use was therefore increased by 3.8% (95% CI − 0.43 to 0.21; *p* = 0.36).

The assessed level of language proficiency of caregivers differed from the self-reported level of language proficiency. Of the study participants in the interview whose language proficiency was screened as limited, 77.1% estimated their language proficiency level as intermediate. More than half of the LLP families who did not receive an interpreter and participated in the interview reported, that they would have liked an interpreter during the consultation.

**Conclusions:** Interpreter services are largely underused during health encounters with LLP families. Relying on caregivers´ self-assessed language proficiency and their active request for an interpreter is not sufficient to ensure safe communication during health encounters. Systematic screening of language proficiency and standardized management of LLP families is feasible and needed at health care facilities to ensure equitable care. Further studies are needed to analyze personal and institutional barriers to interpreter use and find interventions to sustainably increase the use of interpreter services for LLP families.

**Supplementary Information:**

The online version contains supplementary material available at 10.1186/s12913-022-08771-z.

## What is known

- Numbers of patients presenting to health care facilities with limited language proficiency are increasing. Multiple studies indicate that there is an underuse of professional interpreters in health care facilities.
- Impaired communication causes inequity in health care and decreases the quality of its provision.

## What is new

- This is the first study systematically assessing the local language proficiency of caregivers presenting to a pediatric emergency department.
- The majority of the identified families with limited language proficiency did not receive an interpreter. Caregivers with limited language proficiency frequently overestimated their language skills.

## Introduction

With every seventh person no longer living in the place where she or he was born, migration is a growing global reality [1]. Despite ongoing global travel restrictions since 2020, the number of forcibly displaced migrants has increased by the end of 2021 to 84 million people [2]. Persons arriving in host countries with limited local language proficiency (LLP) are therefore a growing population [3]. With the war in Ukraine and 6.6 million people forced to leave their country by the end of May 2022, the issue is becoming even more relevant for European host countries [4]. Many studies highlight that this growing population is at risk of facing inequities in access to healthcare [5–8]. Successful communication between patients and care providers is essential for good quality of care, especially in emergency medicine. Without a mutual understanding between doctor and patient, adequate treatment is not possible. The use of professional interpreters is known to improve quality of care and increase patients’ satisfaction [5, 8–12]. Multiple studies indicate that there is an underuse of interpreters in health care facilities [5, 13–17]. The main reasons for not using professional interpreters are assumed insufficient financial coverage, perceived lack of time of health care workers and lack of knowledge on how to organize professional interpreters [18–20]. Better access to interpreter services, guidelines on interpreter use, and staff training are essential to ensure good quality care for LLP patients [5, 13–17].

Interventions to successfully increase the use of professional interpreters exist [9, 21–23], but are still rare. Several studies highlight the need for simple guidelines and tools to systemically assess the need for interpreters and to facilitate the additional administrative work required to book them [13, 16, 19, 24].

Research on the use of interpreter services at Swiss hospitals is rare. A recently published study on language barriers in primary care using Switzerland as a case study as well as a cross-sectional national survey on how Swiss hospitals address the problem of language barriers in health care show the importance of the topic in the country [25, 26]. Doctors rarely used professional interpreters, and amateur translators such as patients’ family members or friends were more commonly used when language barriers appeared. In some cases, minors were used to provide medical interpretation [25]. This practice is known to be unprofessional and can have severe negative consequences [26–28]. On an ethical-legal level, interpreting services provided by minors, family members or acquaintances are not justifiable [29, 30] and even prohibited by Section 1557 of the Affordable Care Act [31]. In the U. S, Australia and Norway it is a patients civil right to receive an interpreter. Hospitals and doctors have been sued for negative outcomes, thus the provision of interpreters has increased. To improve the use of interpreters, adequate financing and awareness raising within health care facilities were described as most important [20]. A systematic review of hospital interventions published in 2019 including 19 studies highlights that evidence on how to improve health care for LLP patients is scarce. With our study, we thus want to contribute to reducing the large gap in existing evidence in efforts to improve health care of LPP patients [32].

The aim of our study was to systemically assess and improve the use of interpreter services at a pediatric emergency department (ED) in Switzerland taking into account the key aspects for a successful intervention from previous studies.

## Material and methods

### Study area

The study was conducted at the pediatric ED of the University Hospital of Bern, Switzerland. This institution provides the full range of tertiary emergency care to children and adolescents aged 0 to 16 years including surgical, traumatological and pediatric conditions. It provided emergency care to 23,274 patients in 2021. The hospital is part of the Swiss Hospitals for Equity Network [33] since 2021. A phone interpreter service is available at the facility around the clock. It is free of charge for patients and the costs are covered by the department.

### Study design

**Fig. 1:**
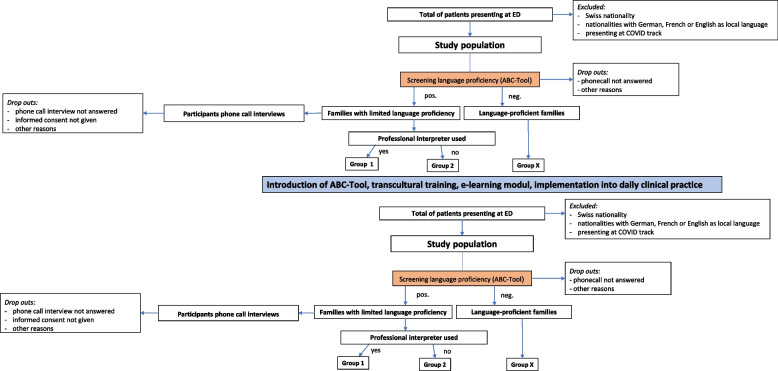
Flowchart depicting the study design

This study was a pre- and post-intervention study analyzing the use of interpreter services for LLP families (Fig. 1). It included families presenting to the ED between April 1st and June 30th 2021 (pre-intervention period) and between October 1st and December 5th 2021 (post-intervention period).

During the 3 months between the data collection periods, a package of interventions was implemented in the ED. The primary outcome was defined as the proportion of LLP families that received an interpreter before and after the package of interventions (Fig. 1). Secondary outcomes were the comparison of the self-reported versus the assessed language proficiency of caregivers, and their knowledge about the interpreter service.

### Study population

**Fig. 2:**
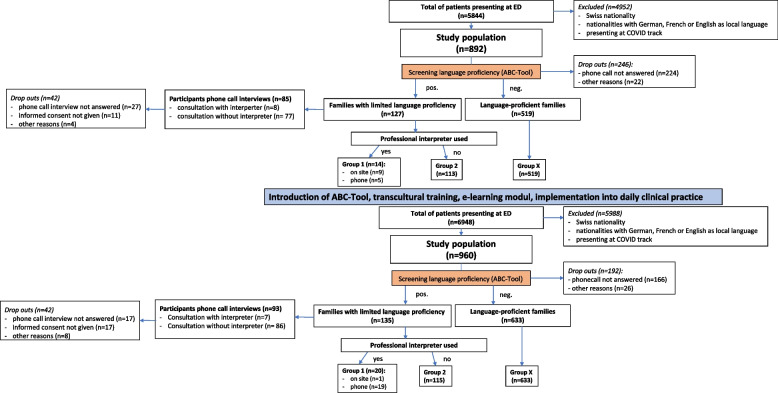
Flowchart depicting the process of inclusion of the study population

From all patients presenting to the ED during the data collection periods, administrative health records were screened and those fulfilling all inclusion criteria were identified. Inclusion criteria were: i) nationality with national language other than German, French or English ii) not presenting on the COVID-19 track. Within 1 week after the consultation, all patients fulfilling these two criteria were systematically screened for their caregiver’s language proficiency by two members of the study team fluent in English, French and German. This was done by phone call interviews with the person who had accompanied the patient during the consultation. In the case of two caregivers present at the consultation, the one with better language skills was screened. A score, validated for the classification of language proficiency was used ranging from A1 (very limited language proficiency) to C2 (excellent language proficiency) [34]. Caregivers with a language level of A1 or A2 (limited language proficiency) or those asking for an interpreter by themselves were defined as LLP families. Patients with a good language proficiency ≥ level B1, were defined as language-proficient families. Those not answering several phone calls or not giving informed consent for the screening were excluded from the final analysis (Fig. 2).

### Intervention

With the aim of increasing the use of interpreter services at the pediatric ED, a package of different activities was provided to the entire emergency team between July 1st and September 30th 2021. It consisted of the following three parts:

#### Blended transcultural training

**Fig. 3:**
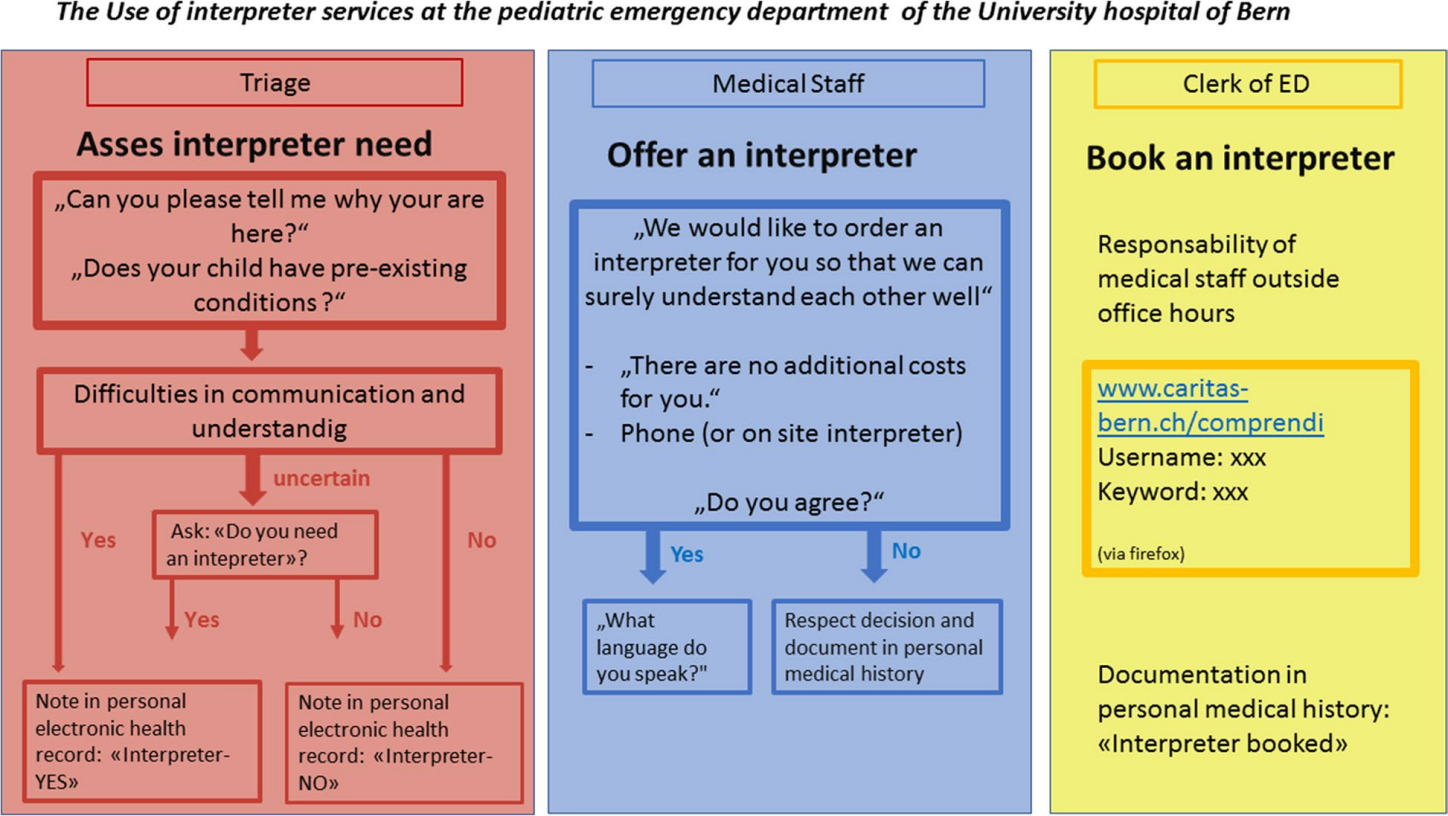
“ABC-Tool” adapted to the use at the pediatric emergency department of the University hospital of Bern (English version) [35]

All health workers were asked to complete the national department of health’s official e-learning module of about 1 h on transcultural competence in healthcare. The in-person part of the transcultural training was conducted on four different dates for all medical staff. The training took 2 h and was conducted by the study team and an expert on intercultural communication from the Swiss Red Cross. The training’s first part focused on awareness raising and on equality and equity in healthcare. It emphasized the ability to communicate as a precondition for equity. It also provided practical instructions and discussions about how to use the ABC screening tool to assess the need for interpreter services in all LLP families at the point of triage and how to order the interpreter services (Fig. 3).

#### Continuous awareness raising

To keep the awareness up and to integrate the new skills into daily routine, several reminders were placed in the departments’ weekly news. The topic was the theme of the month in the form of a visual display of information at the workplace (mid-September-mid-October 2021). Finally, the topic was highlighted during team meetings and the information was integrated in the regular orientation program for new health workers at the department.

#### Introduction of a language proficiency pathway

Originally created at the Western Sydney Local Health District, the ABC-tool is a systematic tool to detect the need for a professional interpreter [35]. The tool was adapted to the local context by the study team (Fig. 3). The health workers at triage categorized individuals into different language competency groups. If the caregiver was comfortable answering the screening-questions, the family was classified as language proficient. If the caregiver did not understand or was not able to answer the questions, the family was classified as LLP. “Interpreter-yes” was noted in the personal electronic health record. If the health worker at triage was not sure about the comprehension and communication skills, the caregiver was asked if he or she wanted an interpreter. If the answer was “yes”, “Interpreter-yes” was also noted in the personal electronic health record. After triage, the responsible physician or nurse actively offered and booked an interpreter online for families with a noted “yes” in the record or also if communication problems had arisen in the meantime. According to the ABC-Tool, the decision to use a professional interpreter is made at the point of triage. However, if language difficulties arise during the consultation, any health care provider can still order an interpreter at any time.

### Data collection and analysis

The following variables were extracted from administrative health records: nationality, age, gender and date of visit. The data on interpreter use was extracted from the electronic bill of interpreter services every month, including the intervention period. Two members of the study team were trained in the use of the validated language proficiency classification of the Goethe institute [34]. They performed phone call screenings with the families meeting the inclusion criteria. The families identified as those with LLP were called with a professional interpreter and asked for consent to participate in the study. From those agreeing to participate, the following variables were collected during a phone interview: native language, assessed language proficiency, self-reported language proficiency and details about the interpreter use. Deidentified data was transferred to a REDcap-database (Vanderbilt University/ Version 11.1.42022). STATA (Stata/MP Version 16.1. 2020) was used for the statistical analysis and generation of graphs.

### Ethics approval

We confirm that all experimental protocols were reviewed by the Ethics Committee of Bern (Study- Nbr: Req-2021-00251) and approved as quality improvement project.

## Results

### Intervention

At the time of intervention, the ED team consisted of 78 medical staff members in total. All of the staff members who completed some form of didactic training were medical front-line providers: 29/78 were physicians, 49/78 were nurses including triage staff. Two-thirds (62%; 48/78) of the team participated either in the e-learning module or in the transcultural in-person training, 17% (13/78) completed both trainings. All were reached by the awareness raising activities.

### Characteristics of the study population

A total of 1582 visits of families meeting the inclusion criteria were identified in the 157-day observation period and 1414 could be screened for their language proficiency. Of those, 262 (18.5%) screened positive (visits of LLP families) and 1152 (81.5%) screened negative (visits of language-proficient families) (Fig. 2).

About one-third of LLP families originated from Syria and Eritrea (19.1%; 15.6%). Most frequent nationalities in language-proficient families were Portuguese (9.2%) and Macedonian (8.6%). Arabic and Tigrinya (39.2%; 28.9%) were the most common languages amongst LLP families, whereas Portuguese and Albanian (19.1%; 34.6%) were most common amongst language-proficient families (Table 1).

**Table 1:** Baseline characteristics of study population

	***Pre-intervention***						***Post-intervention***					
Characteristics	Study population (all families) ***n*** = 892^**a**^		Families with limited language proficiency (ABC positive) ***n*** = 127^**a**^		Language-proficient families (ABC negative) ***n*** = 519^**a**^		Study population (all families) ***n*** = 960^**a**^		Families with limited language proficiency (ABC positive) ***n*** = 135^**a**^		Language-proficient families (ABC negative) ***n*** = 633^**a**^	
	n	IQR/%	n	IQR/%	n	IQR/%	n	IQR/%	n	IQR/%	n	IQR/%
Median age child (years)	4.61	1.68–9.38	5.63	2.09–9.22	4.12	1.62–9.15	3.69	1.65–7.25	3.2	1.59–6.48	3.79	1.72–7.39
Male gender child	489	54.8	74	58.3	290	55.9	520	54.2	66	48.9	339	53.6
Female gender child	403	45.2	53	41.7	229	44.1	440	45.8	69	51.1	294	46.4
**Most frequent nationalities**	PT(*n* = 86)	9.6	SY (*n* = 30)	23.6	PT (*n* = 54)	10.4	ER (*n* = 96)	10.0	ER (*n* = 25)	18.5	MK(*n* = 57)	9.0
	IT (*n* = 77)	8.6	ER (*n* = 16)	12.6	KS (*n* = 47)	9.1	MK (*n* = 77)	8.0	SY (*n* = 20)	14.8	ER (*n* = 55)	8.7
	KS (*n* = 76)	8.5	PT (*n* = 13)	10.2	MK (*n* = 45)	8.7	PT (*n* = 76)	7.9	AF (n = 13)	9.6	PT (*n* = 52)	8.2
	MK (*n* = 65	7.3	LK (*n* = 12)	9.5	IT (*n* = 42)	8.1	IT (*n* = 73)	7.6	IT (*n* = 12)	8.9	IT (*n* = 50)	7.9
**Most frequent native languages**	Albanian (*n* = 118)	17.2	Arabic (*n* = 30)	23.6	Albanian (*n* = 109)	19.5	Albanian (*n* = 117)	13.9	Tigrinya (*n* = 25)	17.1	Albanian (*n* = 105)	15.1
	Portuguese (*n* = 73)	10.6	Tigrinya (*n* = 15)	11.8	Portuguese (*n* = 59)	10.6	Arabic (*n* = 72)	8.6	Arabic (*n* = 23)	15.6	Portugese (*n* = 59)	8.5
	Arabic (*n* = 65)	9.5	Portuguese (*n* = 14)	11.0	Arabic (*n* = 35)	6.3	Tigrynia (*n* = 70)	8.3	Italian (*n* = 14)	9.6	Kurdish (*n* = 52)	7.5
	Kurdish (*n* = 41)	6.0	Tamil (*n* = 12)	7.1	Italian (*n* = 35)	6.3	Portuguese (*n* = 64)	7.6	Albanian (*n* = 12)	8.2	Arabic (*n* = 49)	7.1
**Estimated language proficiency**												
- A1	50	5.6	50	39.4	0	0	49	5.1	49	36.3	0	0
- A2	72	8.1	72	56.7	0	0	78	8.1	78	57.8	0	0
- B1	153	17.1	0	0	153	29.5	197	20.5	1	0.7	196	31.0
- B2	145	16.2	0	0	145	27.9	206	21.5	0	0	206	32.5
- C1	126	14.1	0	0	126	24.3	116	12.1	0	0	116	18.3
- C2	95	10.7	0	0	95	18.3	112	11.7	0	0	112	17.7
- missing	251	28.1	5	3.9	0	0	202	21.0	7	5.2	3	0.5
**Interpreter use**	**14**	**2.8**	**14**	**11.0**	0	0	**20**	**2.1**	**20**	**14.8**	0	0
- on site	9	64.3	9	64.3	0	0	1	5.0	1	5.0	0	0
- phone	5	35.7	5	35.7	0	0	19	95.0	19	95.0	0	0

^a^*n* = number of visits not number of families, *AF* Afghanistan, *ER* Eritrea, *IT* Italy, *KS* Kosovo, *LK* Sri Lanka, *MK* Macedonia, *PT* Portugal, *SY* Syria, A1 very limited language proficiency, A2 limited language proficiency, B1 low intermediate language proficiency, B2 high intermediate language proficiency, C1 high language proficiency, C2 very high language proficiency

### Interpreter use

The proportion of LLP families who received an interpreter was 11.0% (14/127) in the pre-intervention period compared to 14.8% (20/135) in the post-intervention period. The interpreter use was therefore increased by 3.8% through the intervention, which was not statistically significant (difference in proportions 0.038, 95% CI − 0.43 to 0.21; *p* = 0.36). In the pre-intervention period, a professional interpreter was used 14 times, five times as phone interpreters and nine times as on-site interpreters. In the post-intervention period, an interpreter was used 20 times. 19 times it was a phone interpreter and one time an on-site interpreter. None of the language-proficient families received an interpreter.

### Language proficiency

Of those defined as LLP families, 37.8% (99/262) where classified as level A1 (very limited language proficiency). 57.3% (150/262) were classified as level A2 (limited language proficiency). In the group of language-proficient families, level B1 (low intermediate language proficiency) pre-intervention (29.5% (153/519)) and level B2 (high intermediate language proficiency) post-intervention (32.5% (206/633)) were most common (Table 1).

**Fig. 4:**
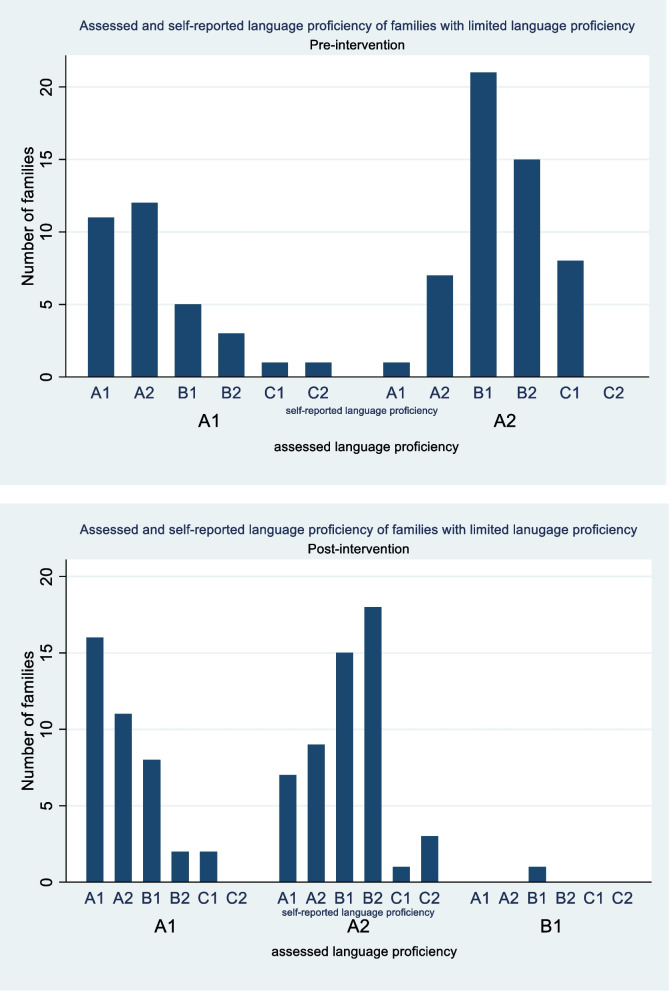
Assessed and self-reported language proficiency of families with limited language proficiency. A1 = very limited language proficiency; A2 = limited language proficiency; B1 = low intermediate language proficiency; B2 = high intermediate language proficiency; C1 = high language proficiency; C2 = excellent language proficiency

The self-reported language proficiency in the phone call interviews with positively screened families differed from the investigator’s language assessment (Fig. 4). 178 families were asked about their self-estimated language proficiency. Those with an estimated language proficiency of A1 most often correctly assessed themselves as level A1 (37.2% ((27/72)). But nonetheless 30.6% (22/72) of families assessed as A1 level reported a self-estimated language proficiency ≥B1. Among the assessed A2 level group, the proportion of self-reported language proficiency ≥B1 was even higher at 77.1% (81/105; most frequently B1 (34.3% (36/105)).

### Communication

Of those LLP families who participated in the phone call interview and did not receive an interpreter during consultation, more than half (54.0% (88/163)) reported difficulties in communication and understanding. 35.6% (58/163) reported using a nonprofessional interpreter during the consultation (Table 2). A total of 47.8% (22/46) of LLP families who used a nonprofessional interpreter during the consultation and answered the question about the interpreter’s age reported, that a minor below 18 years of age translated during the consultation. More than half of the families (54.0% (88/163)) who did not receive an interpreter and participated in the phone interview reported, that they would have liked and interpreter during the consultation. Only a quarter of participating families (24.7% (22/178)) knew about their possibility to a free of charge interpreter service. A more detailed analyses of this topic can be found in a mixed method study which was done simultaneously to this research study (Gmünder M et al. The satisfaction of migrant families with the quality of paediatric emergency care related to the use of professional interpreter services - a mixed methods study. Unpublished work).

**Table 2:** Perspective of families with limited language proficiency

	Perceived language barrier pre-intervention (*n* = 77)/ post-intervention (*n* = 86)^ab^				Interpreter desired pre-intervention (*n* = 77)/ post-intervention (*n* = 86)^ab^				Knowledge about interpreter entitlement pre-intervention (*n* = 85)/ post-intervention (*n* = 93)^ac^				Communication with non-professional interpreter pre-intervention (*n* = 77)/ post-intervention (*n* = 86)^ab^			
	*Yes*		*No*		*Yes*		*No*		*Yes*		*No*		*Yes*		*No*	
	n	%	n	%	n	%	n	%	n	%	n	%	n	%	n	%
***Pre-Intervention***	40	51.9	37	48.1	38	49.4	39	50.6	22	25.9	63	74.1	27	35.1	50	64.9
***Post-intervention***	48	55.8	38	44.2	50	58.1	36	41.9	24	25.8	69	74.2	31	36.0	55	64.0

^ab^ Answers of families with limited language proficiency who participated in a phone call interview and didn’t receive an interpreter

^ac^ Answers of all families with limited language proficiency who participated in a phone call interview

## Discussion

This interventional study analyzed the use of interpreters before and after the implementation of a language proficiency pathway for LLP families. The pediatric ED of the University of Bern is the first hospital department in Switzerland with a structured screening for language proficiency. The study showed that a very high number of LLP families did not receive an interpreter. This likely leads to lower quality of care and ultimately a gap in health equity as demonstrated by other studies [5, 15, 17, 25].

Our intervention slightly, but not significantly, increased the use of interpreter services. There was an important increase in interpreter use during the intervention period but the effect could not be maintained (Supplementary Fig. 1). A Swiss study on barriers to the use of professional interpreters in primary healthcare highlighted a lack of knowledge on how to organize professional interpreters and insufficient financial coverage as main reasons for not using professional interpreters [20]. Other studies assessing quality improvement approaches [9, 21–23, 36] highlight the convenient accessibility of phone interpreters [9], standardized protocols [36] and appropriate documentation [21] as key components to success. The design of the study intervention took these factors into account by ensuring free, 24-hour access to phone interpreter services, implementing a clear, standardized protocol and documentation, and having an administrative clerk to facilitate the process. Yet the intervention did not significantly increase the use of interpreters. One potential reason for the limited effect was a peak of patients after the relaxation of the measures of Covid-19 crisis, to be considered as rebound phenomenon, during the post-intervention period resulting in an unexpectedly high patient volume (Complementary Fig. 1), resulting in high levels of stress and limited time per consultation. Concerns about time-consuming language interpretation were often mentioned by the medical staff during informal discussions in the transcultural training. As shown by pediatric studies from the United States and Sweden, perceived lack of time and high stress levels often hinder staff from using interpreters [18].

Despite the perception of time-consuming interpreter consultations, studies show that the use of interpreter services does not prolong or shorten the length of stay in the ED [37]. Optimizing the processes of the implemented pathway and ongoing awareness raising to change the perception of time-consuming interpreter consultations might help to further increase the use of interpreters. Technology based interpretation may, in the future, help improve communication in a time-efficient way [21]. A pilot project conducted at four different Swiss hospitals using an artificial intelligence based interpretation device has already shown promising preliminary results in health worker satisfaction [33]. Providing easy access to professional interpreters, immediately available to providers anytime, is likely to increase the use of interpretation services as shown in a quality improvement project on a medicine ward which provided a dual-handset interpreter telephone at the bedside of every patient with LLP [9].

There was a remarkable discrepancy between the assessed language proficiency during screening and the self-reported language proficiency with caregivers overestimating their language skills. This finding is in line with a study conducted in Montreal. The results showed that by asking for language preference alone, the need for an interpreter in many LLP families would remain undetected [38].

Furthermore, only a small proportion of LLP families knew about the option to get a timely and free of charge interpreter service whereas most of the study participants would have liked an interpreter-assisted consultation. Using family members, and often children, as interpreters during the health care visit was not a deliberate choice for most of them but a necessity. Staff accepted this solution out of convenience although it is known that professional interpreters are less likely to make errors with potentially harmful consequences than ad hoc interpreters [10, 39]. Interpretation by minors impose additional legal and ethical challenges. This result highlights the importance of defining and reinforcing the use of professional interpreters as an institutional standard. Networks such as “Swiss Hospital for Equity” supported by the federal office of public health can help to fill gaps such as the underuse of interpreters on a national level [33].

In addition, LLP families need to be informed about their right to receive professional interpretation and the advantages of using them. Including LLP caregivers in the study team might help to further improve the design of interventions aiming to improve the use of interpreter services. An area of future research could be to assess how providers’ self-reported language proficiency affects their utilization of interpreters.

### Strengths and limitations

This study has several limitations. In addition to the higher workload during the post-intervention period, the shiftwork, the high turnover of residents and the fact that the majority of staff is working part time, reduced the number of people participating at the in-person training, which was a core component of the intervention. In this study, the language screening was done via phone call which might create slightly different results compared to an in-person screening at the point of triage. As a single-site study findings of this study may not be generalizable to other sites. An analysis to show whether the educational activity increased staff awareness of the importance to use trained interpreters and increased sensitivity to recognize the need for an interpreter was beyond the scope of this study. However an internal online-survey showed positive results in both domains.

The greatest strength of this study was the systematic implementation of a language proficiency pathway as institutional standard. By introducing a systematic language screening, this study detected an important gap in the use of professional interpreters, likely to be present at many healthcare facilities across the country. The pathway could serve as a good practice example for other hospitals to facilitate more equitable communication in health care in Switzerland.

## Conclusion

Interpreter services are largely underused during health encounters with LLP families. Relying on caregivers´ self-assessment in language proficiency and their active request for an interpreter is not sufficient to ensure safe communication during health encounters. Systematic screening of language proficiency and standardized management of LLP families is feasible and needed at health care facilities to ensure equitable care. Further studies are needed to analyze personal and institutional barriers to interpreter use and find interventions to sustainably increase the use of interpreter services for LLP families.

## Supplementary Information

**Additional file 1: Supplementary Figure** **1.** Total of patients visiting the pediatric emergency department of the University hospital of Bern, Switzerland and total interpreter use during the study period.

## Data Availability

The datasets used and/or analyzed during the current study are available from the corresponding author on reasonable request.

## Abbreviations

ED: Emergency department
LLP: Limited language proficiency
