# Correction: The use of intercultural interpreter services at a pediatric emergency department in Switzerland

**DOI:** 10.1186/s12913-022-08964-6

**Published:** 2022-12-19

**Authors:** Sina Buser, Noemi Gessler, Myriam Gmuender, Ursula Feuz, Anne Jachmann, Jabeen Fayyaz, Kristina Keitel, Julia Brandenberger

**Affiliations:** 1 Division of Pediatric Emergency Medicine, Department of Paediatrics, Inselspital, Bern University Hospital, University of Bern, Bern, Switzerland; 2 Emergency Department, Inselspital, Bern University Hospital, University of Bern, Bern, Switzerland; 3 Division of Pediatric Emergency Medicine, Hospital for Sick Children, Toronto, Ontario Canada; 4 Edwin S.H. Leong Centre for Healthy Children, University of Toronto, Toronto, ON Canada

**Correction: BMC Health Serv Res 22, 1365 (2022)**

**https://doi.org/10.1186/s12913-022-08771-z**

Following publication of the original article [1], the authors identified an error in the author names. The given names and family names of all authors were erroneously transposed.

The incorrect author names are: Buser Sina, Gessler Noemi, Gmuender Myriam, Feuz Ursula, Jachmann Anne, Fayyaz Jabeen, Keitel Kristina and Brandenberger Julia

The correct author names are: Sina Buser, Noemi Gessler, Myriam Gmuender, Ursula Feuz, Anne Jachmann, Jabeen Fayyaz, Kristina Keitel and Julia Brandenberger

The author group has been updated above and the original article [1] has been corrected.
