# Supplementary material for: The use of intercultural interpreter services at a pediatric emergency department in Switzerland
Source: BMC Health Serv Res. 2022 Nov 17;22:1365. doi: 10.1186/s12913-022-08771-z (PMC9670076; doi:10.1186/s12913-022-08771-z)

*Supplementary Fig. 1:* Summary of all patients visiting the pediatric ED and total interpreter use during the study period


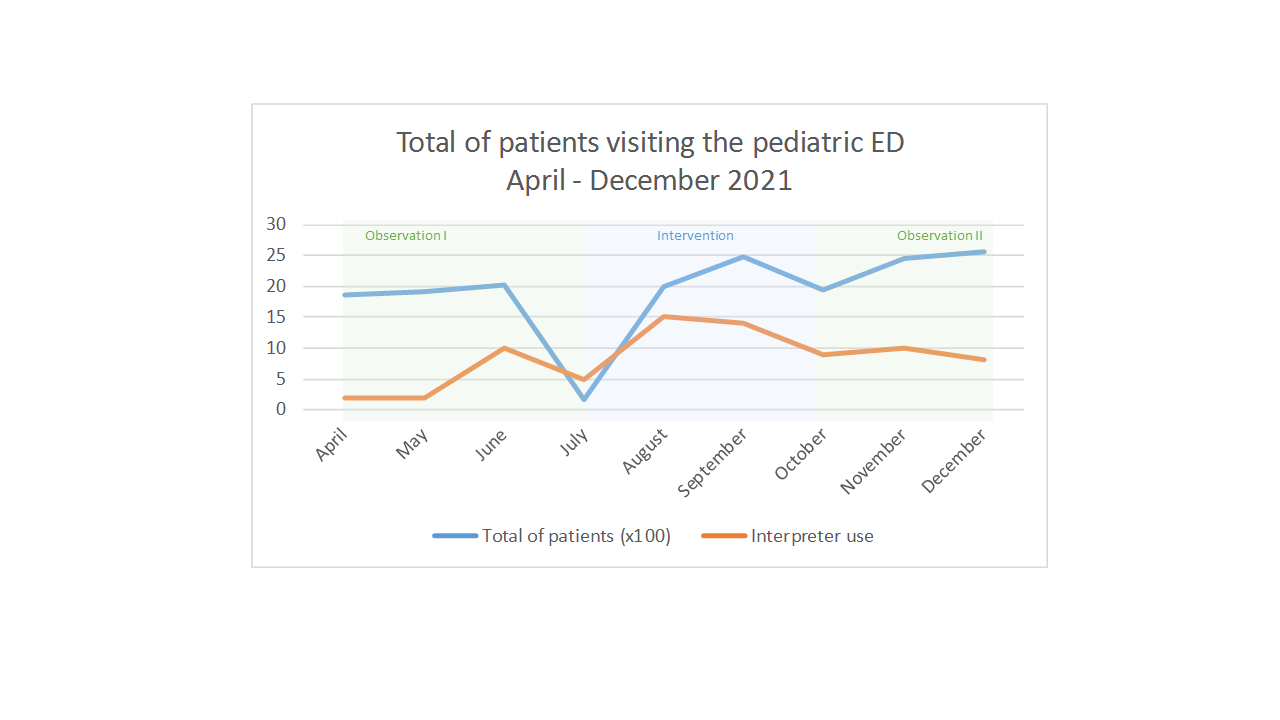

Supplement: Supplementary file 1 — Additional file 1: Supplementary Figure 1. Total of patients visiting the pediatric emergency department of the University hospital of Bern, Switzerland and total interpreter use during the study period. [file 12913_2022_8771_MOESM1_ESM.docx]
